# Application of Regent mechanical valve in patients with small aortic annulus: 3-year follow-up

**DOI:** 10.1186/1749-8090-7-88

**Published:** 2012-09-21

**Authors:** Dong Zhao, Chunsheng Wang, Tao Hong, Cuizhen Pan, Changfa Guo

**Affiliations:** 1 Department of Cardiac Surgery, Zhongshan Hospital Fudan University & Shanghai Institute of Cardiovascular Diseases, Shanghai, 200032, People's Republic of China; 2 Department of Echocardiograph, Shanghai Institute of Cardiovascular Diseases, Shanghai, 200032, People’s Republic of China

**Keywords:** Small aortic annulus, St.Jude Medical Regent mechanical valve, Left cardiac function, Prothesis-Patient Mismatch

## Abstract

**Background:**

Aortic valve replacement (AVR) with a small aortic annulus is always challenging for the cardiac surgeon. In this study, we sought to evaluate the midterm performance of implantation with a 17-mm or 19-mm St. Jude Medical Regent (SJM Regent) mechanical valve in retrospective consecutive cohort of patients with small aortic annulus (diameter ≤ 19 mm).

**Methods:**

From January 2008 to April 2011, 40 patients (31 female, mean age = 47.2 ± 5.8 years) with small aortic annulus (≤19 mm in diameter) underwent aortic valve replacement with a 17-mm or 19-mm St. Jude Medical Regent (SJM Regent) mechanical valve. Preoperative mean body surface area, New York Heart Association class, and mean aortic annulus were 1.61 ± 0.26 m^2^, 3.2 ± 0.4, and 18 ± 1.4 mm respectively. Patients were divided into two groups, according to the implantation of 17 mm SJM Regent mechanical valve (group 1, n = 18) or 19 mm SJM Regent valve (group 2, n = 22). All patients underwent echocardiography examination preoperatively and at one year post-operation.

**Results:**

There were no early deaths in either group. Follow-up time averaged 36 ± 17.6 months. The mean postoperative New York Heart Association class was 1.3 ± 0.6 (p < 0.001). By echocardiography, in group 1, the left ventricular ejection fraction (LVEF), left ventricular fraction shortening (LVFS), and the indexed effective orifice area (EOAI) increased from 43.7% ± 11.6%, 27.3% ± 7.6%, and 0.70 ± 0.06 cm^2^/m^2^ to 69.8 ± 9.3%, 41.4 ± 8.3%, and 0.92 ± 0.10 cm^2^/m^2^ respectively (*P* < 0.05), while the left ventricular mass index (LVMI), and the aortic transvalvular pressure gradient decreased from 116.4 ± 25.4 g/m^2^, 46.1 ± 8.5 mmHg to 86.7 ± 18.2 g/m^2^ , 13.7 ± 5.2 mmHg respectively. In group 2, the LVEF, LVFS and EOAI increased from 45.9% ± 9.7%, 30.7% ± 8.0%, and 0.81 ± 0.09 cm^2^/m^2^ to 77.4% ± 9.7%, 44.5% ± 9.6%, and 1.27 ± 0.11 cm^2^/m^2^ respectively, while the LVMI, and the aortic transvalvular pressure gradient decreased from 118.3 ± 27.6 g/m^2^, 44.0 ± 6.7 mmHg to 80.1 ± 19.7 g/m^2^, 10.8 ± 4.1 mmHg as well. The prevalence of PPM was documented in 2 patients in Group 1.

**Conclusions:**

Patients with small aortic annulus and body surface area, experienced satisfactory clinical improvement after aortic valve replacement with modern SJM Regent bileaflet prostheses.

## Background

Aortic valve replacement (AVR) with a small aortic annulus is always challenging for the cardiac surgeon. Traditional small mechanical valves, especially if implanted in patients with big body surface area, may show high aortic transvalvular pressure gradients, the so-called patient–prosthesis mismatch (PPM) phenomenon [1]. Several surgical strategies can be applied to avoid PPM, among them aortic root enlargement procedure seems a preventive potential strategy to minimize mismatch, since it showed good early and long-term results in experienced centers [2]. However, this approach is complex and time-consuming that may cause serious bleeding and infection, and can be difficult to perform in patients with a small, calcified aortic annulus and root.

On the other hand, modern St.Jude Medical Regent (SJM Regent) mechanical valve is much more favored for the unique design and the bigger effective orifice area (EOA). By shifting the sewing cuff and the retaining ring to the supra-annular position, the SJM Regent valve achieve a greater geometric orifice for a fixed diameter, which may prevent PPM phenomenon in patients with small aortic annulus as well [3-5]. In our present study, we sought to review the midterm performance of implantation with a 17-mm or 19-mm SJM Regent mechanical valve in consecutive patients with small aortic annulus (diameter ≤ 19 mm), and evaluate whether it is a preventive strategy to eliminate PPM.

## Methods

### Patients

From January 2008 to April 2011, 1946 AVR procedures with mechanical prostheses were performed in our hospital. A 17-mm or a 19-mm SJM Regent was implanted in 54 (2.8%) patients. Of those 54 patients, 14 patients were excluded because of the big aortic annulus (>19 mm in diameter); thus, only 40 patients entered the study. Those 40 patients (31 female versus 9 male) with small aortic annulus (≤19 mm in diameter) underwent aortic valve replacement with a 17-mm or 19-mm St. Jude Medical Regent (SJM Regent) mechanical valve. The mean age was 47.2 ± 5.8 years (ranging from 17 to 69 years), and the body surface area was 1.61 ± 0.26 m^2^ (ranging from 1.51 to 1.77 m^2^). Preoperative average New York Heart Association class was 3.2 ± 0.4 with 32 patients of grade III and 8 patients of grade IV. Aortic valve characteristics were summarized in Table 1. Patients were divided into two groups, according to the implantation of 17 mm SJM Regent mechanical valve (group 1, n = 18) or 19 mm SJM Regent valve (group 2, n = 22). The calculation formulae of body surface area (BSA) were:

(1) $$\begin{array}{l} \text{Male}:\text{S}=0.0057\times \text{height}\left(\text{cm}\right)+0.0121\times \text{weight}\left(\text{kg}\right)+0.0882;\\ \text{Female}:\text{S}=0.0073\times \text{height}\left(\text{cm}\right)+0.0127\times \text{weight}\;\left(\text{kg}\right)-0.2106\text{.} \end{array}$$

**Table 1:** Aortic valve characteristics

	**Group 1 (17 mm Regent, n = 18)**	**Group 2 (19 mm Regent, n = 22)**	***P*** **value**
Aortic stenosis (congenital)	2 (11.1%)	3 (13.6%)	NS
Aortic stenosis (degenerative)	4 (22.2%)	3 (13.6%)	NS
Aortic insufficiency (rheumatic)	8 (44.4%)	10 (45.5%)	NS
Aortic stenosis and insufficiency (rheumatic)	3 (16.7%)	4 (18.2%)	NS
SBE	1 (5.6%)	2 (9.1%)	NS

The surgical procedures were performed through a midline sternotomy under cardiopulmonary bypass and mild hypothermia. Myocardial protection was achieved with cold blood cardioplegia perfusion into coronary arteries or the aortic root, and the surface of heart was covered with ice. Valve prostheses were implanted at the valve ring level with non-everting interrupted mattress sutures. After the second postoperative day, patients received oral anticoagulation with sodium warfarin at daily updated dosages according to international normalized ratios. The target international normalized ratio value was in accordance with American Heart Association guidelines.

### Echocardiography

A complete M-mode, two-dimensional, and Doppler evaluation was performed at the probe frequency of 2.5/2.7 MHZ using commercially available ultrasonographic equipment (HP Sonos5500) in Zhongshan Hospital, Fudan University. Left ventricular (LV) end-diastolic and end-systolic diameters and volumes, as well as left ventricular ejection fraction (LVEF), left ventricular fraction shortening (LVFS), were calculated using the area-length method. Left ventricular mass index (LVMI) was calculated from Reichek’s formula. As regard to aortic valve evaluation, we measured indexed aortic valve area (EOAI), aortic annulus diameter, and the aortic transvalvular pressure gradient. Echocardiographic evaluation with the same variables was repeated at follow-up.

### Follow-ups

All patients were followed in the outpatient center with clinical visits and echocardiography performed on a 6-month basis. A telephone interview was required only for patients with follow-up visits in excess of 6 months or for those lost to ambulatory follow-up. The follow-up was 100% complete, and the mean time to last follow-up was 36 ± 17.6 months (ranging from 12 to 51 months).

### Statistics

Statistics were calculated using stata 7.0. The continuous varieties were expressed as average ± standard deviation, the Wilcoxon method was used to test the difference between two continuous varieties, and *t*-test was used to test paired samples. P < 0.05 means there is statistical differences, and P < 0.01 represents significant statistical differences.

## Results

### Clinical results and follow-up

Operative details were shown in Table 2. An AVR procedure was performed in all 40 patients. Concomitant performed procedures included MV surgery, TV surgery, and CABG, and there were no difference between the two groups. Mean crossclamp time was similar between treatment groups (1 vs 2) (45 ± 6 minutes vs 51 ± 4 minutes; *P* = .6), as was total bypass time (78 ± 6 minutes vs 81 ± 7 minutes; P = .54).

**Table 2:** Concomitant operative characteristics

	**Group 1 (17 mm Regent, n = 18)**	**Group 2 (19 mm Regent, n = 22)**	***P*** **value**
MV surgery	8 (44%)	9 (41%)	NS
TV surgery	6 (33%)	7 (32%)	NS
CABG	2 (11%)	3 (14%)	NS
Total bypass time	78 ± 6 minutes	81 ± 7 minutes	NS
Crossclamp time	45 ± 6 minutes	51 ± 4 minutes	NS

There were no early deaths in either group. One early nonfatal cerebrovascular accident was observed in group 2. No severe arrhythmia, low cardiac output syndrome, pericardial tamponade, respiratory failure, hepatic failure and renal failure were observed. Follow-up time averaged 36 ± 17.6 months. The mean postoperative New York Heart Association class was 1.3 ± 0.6 (p < 0.001), and no further 1-year mortality occurred.

### Evaluation of heart function

All patients underwent echocardiography examination preoperatively and at one year post-operation.

As shown in Table 3, the left cardiac functions of the patients replaced with Regent valve were improved at one year after the operation as compared to pre-operation. By echocardiography, in group 1, the LVEF and LVFS increased from 43.7% ± 11.6%, 27.3% ± 7.6% pre-operatively to 69.8 ± 9.3%, 41.4 ± 8.3% after operation, respectively (*P* < 0.01). In group 2, the LVEF and LVFS increased from 45.9% ± 9.7%, 30.7% ± 8.0%, and to 77.4% ± 9.7%, 44.5% ± 9.6% as well (*P* < 0.01). There was no significant difference between two groups.

**Table 3:** Comparison of cardiac function in two groups

		**LVEF(%)**	**LVSF(%)**	**LVMI(g/m**^**2**^**)**	**Gradient**	**EOAI**
					**(mmHg)**	**(cm**^**2**^**/m**^**2**^**)**
**Group 1**	**Pre**	43.7 ± 11.6	27.3 ± 7.6	116.4 ± 25.4	46.1 ± 8.5	0.70 ± 0.06
	**Post**	69.8 ± 9.3*	41.4 ± 8.3*	86.7 ± 18.2#	13.7 ± 5.2*	0.92 ± 0.10#
**Group 2**	**Pre**	45.9 ± 9.7	30.7 ± 8.0	118.3 ± 27.6	44.0 ± 6.7	0.81 ± 0.09
	**Post**	77.4 ± 9.7*	44.5 ± 9.6*	80.1 ± 19.7#	10.8 ± 4.1*	1.27 ± 0.11#$

Pre: pre-operation; Post: one year post-operation; ^#^ P < 0.05 , ^*^ P < 0.01 as compared to pre-operation, ^$^ P < 0.01, as compared to group 1 post-operation.

In both groups, left ventricular mass regression occurred. In group 1, LVMI decreased from 116.4 ± 25.4 g/m^2^ pre-operatively to 86.7 ± 18.2 g/m^2^ at follow-up (*P* < 0.05), while in group 2, it decreased from118.3 ± 27.6 g/m^2^ pre-operatively to 80.1 ± 19.7 g/m^2^ at follow-up (*P* < 0.05).

Using a 17-mm or 19-mm SJM Regent, also shown in Table 3 in both groups, hemodynamic performance improved as well. In group 1, aortic transvalvular pressure gradient decreased from 46.1 ± 8.5 mmHg to 13.7 ± 5.2 mmHg (*P* < 0.01).In group 2, aortic transvalvular pressure gradient decreased from 44.0 ± 6.7 mmHg to 10.8 ± 4.1 mmHg (*P* < 0.01). The hemodynamic performances in the two groups showed no significant difference (13.7 ± 5.2 mmHg versus 10.8 ± 4.1 mmHg).

On the other hand, though EOAI increased in both groups at one-year follow-up as compared to pre-operation, a 19-mm SJM Regent showed superior EOAI as compared to that in group 1(1.27 ± 0.11 cm^2^/m^2^ versus 0.92 ± 0.10 cm^2^/m^2^, P < 0.01).

### Prothesis-Patient Mismatch

The prevalence of PPM, defined as an indexed effective orifice area (EOA) of less than 0.85 cm^2^/m^2^, was documented in 2 patients (11%, the EOA of the one patient was 0.77 cm^2^/m^2^, the other was 0.83 cm^2^/m^2^) in group 1, and 0 in group 2. These two patients were both male with larger body surface area. However, the presence of PPM did not affect early and mid-term survival or postoperative functional status. A significant reduction of aortic transvalvular pressure gradient occurred in both patients.

## Discussion

PPM, a concept widely accepted by many scholars [1,6-16], refers to a series of complications or potential risks that occurred when the size of a implanted valve prosthesis mismatches the patients’ BSA. Several surgical strategies can be applied to avoid PPM, among them aortic root enlargement procedure seems a preventive potential strategy to minimize mismatch. However, this approach is complex and time-consuming that may cause serious bleeding and infection, and can be difficult to perform in patients with a small, calcified aortic annulus and root. In the present study, we applied modern SJM Regent valves in patients with small aortic annulus, and sought to evaluate whether SJM Regent valve could be an alternate choice to minimize PPM.

It is no doubt that PPM has a significant negative impact on the regression of left ventricular hypertrophy, the improvement of left cardiac function, and a long life survival, as Castro point out [2]. To minimize PPM after AVR, we need to understand the most determinant factors of PPM. In 1997, Sawant pointed out BSA ≥ 1.9 m^2^ as a risk factor for patients replaced with small mechanical valve (outer diameter < 21 mm), and may lead to sudden death [17]. Later in 2000, Pibarot [6] held the view that whether there will be PPM after operation depends on BSA, as well as effective orifice area of the valve prosthesis. More recently, Botzenhardt [18] found out that the ratio of EOA of the valve to BSA (EOAI) is an indicator which is more important than the aortic transvalvular pressure gradient. As they concluded, when EOAI > 0.85 cm^2^/m^2^, no PPM is presented; when the EOAI is between 0.65-0.85 cm^2^/m^2^, a moderate PPM can be seen; and if the EOAI is lower than 0.65 cm^2^/m^2^, the PPM can be severe [6,18]. From this view, given that the BSA of the patient is stable, to minimize PPM after AVR, we need to increase the EOA of the implanted valve.

Nowadays, there are three strategies to increase the EOA of the implanted valve when treating patients with small aortic annulus [19]: (1) aortic annulus enlargement; (2) stentless bioprothesis valve; (3) mechanical valve of same diameter with bigger EOA, including SJM Regent mechanical valve, Carbomedics Top-hat valve, Sorin Slimline valve, and ATS AP valve. Although annulus/root enlargement procedures, as well as stentless valve implantation, performed in experienced centers, showed good early and long-term results [2,20], these two procedures were complex and time-consuming. By shifting the sewing cuff and the retaining ring to the supra-annular position, the SJM Regent possesses a larger EOA. In our present study, most (38/40) patients got a satisfactory heart function improvement and life quality after implantation of a 17 mm or 19 mm Regent valve with two patients of PPM. The good results are persistent with the superior dynamic aspects of Regent valve as others showed. Kon’s research on Regent valve revealed that the blood flows through the valve is laminar, the transvalvular pressure gradient is low, and the EOA and EOAI are satisfying [21]. The report showed that the mean/peak aortic transvalvular pressure gradient were 3/7 mmHg for healthy people at rest and 4/9 mmHg in motion, and the application of Regent valve could meet the standard [17]. Due to the aspect of hemodynamics, Okumiya hold the view that the cardiac indexes of patients replaced with SJM Regent mechanical valve are far more better than those of the patients replaced with conventional mechanical valves [22].

In our present study, using 17 mm or 19 mm Regent valve as prosthesis, we performed the operations with mean crossclamp time of about 50 minutes. This procedure was much simpler as compared to aortic annulus enlargement and implantation of stentless bioprothesis valve. Further, no operation related death or serious postoperative complications were presented in our study. Furthermore, the heart function of all patients (LVEF and LVFS) at one year after operation improved with left ventricular mass regression and decrease of aortic transvalvular pressure gradient. It suggests that using small Regent valve be an alternate choice for cardiac surgeons when facing patients with small aortic annulus.

In our present study, 2 PPMs were documented in our group 1. In our present study, the incidence of PPM is relatively low (only 11% in group-1 and 0% in group-2). According to the information of St Jude Company, the 17 mm Regent valve is suitable for patients with BSA <1.7 m^2^, and 19 mm Regent valve is suitable for patients with BSA < 1.9 m^2^. In our study, the BSA of the two groups was 1.61 ± 0.26 m^2^. Actually, the BSA in group 1 (17 mm Regent) was 1.53 ± 0.12 m^2^ (data not shown). That is perhaps the main reason for our low PPM incidence. Besides, Dr. Minardi G, and Sezai A also showed similar low incidence of PPM to our study (10.5% and 10.3%,respectively) [12,23]. Fortunately, as for these two patients, the presence of PPM did not affect early and 3-year survival or postoperative functional status. Further, a significant reduction of aortic transvalvular pressure gradient occurred in both patients. It might be attributed to that the EOA of the two patients (0.77 cm2/m2 and 0.83 cm^2^/m^2^) were little smaller than 0.85 cm2/m2. The long-term follow-up needs to be carefully monitored.

## Conclusion

In conclusion, for patients with small aortic annulus and body surface area, implantation of SJM Regent bileaflet prostheses would bring along heart function improvement and clinical satisfaction of the patients, and would be a better choice as compared to aortic annulus enlargement.

## Consent

Written informed consent was obtained from the patient for publication of this report and any accompanying images.

## Competing interests

The authors declare that they have no competing interests.

## Authors’contributions

DZ, CG and CSW designed the study. DZ, TH collected the clinical data. CZP performed the echocardiography. DZ drafted the manuscript. CG and CSW did the revision. All authors read and approved the final manuscript.
